# Radiomics Nomogram Analyses for Differentiating Pneumonia and Acute Paraquat Lung Injury

**DOI:** 10.1038/s41598-019-50886-7

**Published:** 2019-10-21

**Authors:** Wang Yanling, Gao Duo, Geng Zuojun, Shi Zhongqiang, Wu Yankai, Lu Shan, Cui Hongying

**Affiliations:** 1 Department of Medical Imaging, Second Hospital of Hebei Medical University, Shijiazhuang, 050000 China; 2 GE Healthcare, Shanghai, 210000 China

**Keywords:** Risk factors, Risk factors, Risk factors, Mathematics and computing, Mathematics and computing

## Abstract

Paraquat poisoning has become a serious public health problem in some Asian countries because of misuse or suicide. We sought to develop and validate a radiomics nomogram incorporating radiomics signature and laboratory bio-markers, for differentiating bacterial pneumonia and acute paraquat lung injury. 180 patients with pneumonia and acute paraquat who underwent CT examinations between December 2014 and October 2017 were retrospectively evaluated for testing and validation. Clinical information including demographic data, clinical symptoms and laboratory test were also recorded. A prediction model was built by using backward logistic regression and presented on a nomogram. The radiomics-based features yielded areas under the receiver operating characteristic curve of 0.870 (95% CI 0.757–0.894), sensitivity of 0.857, specificity of 0.804, positive predictive value of 83.3%, negative predictive value of 0.818 in the primary cohort, while in the validation cohort the model showed similar results (0.865 (95% CI 0.686–0.907), 0.833, 0.792, 81.5%, respectively). The individualized nomogram included radiomics signature, body temperature, nausea and vomiting, and aspartate transaminase. We have developed a radiomics nomogram that combination of the radiomics features and clinical risk factors to differentiate paraquat lung injury and pneumonia for patients with an unclear medical history of exposure to paraquat poisoning, providing appropriate therapy decision support.

## Introduction

Since the 1980s, the risks posed by pesticides have become increasingly concerned in international communities, especially in China. Paraquat is one of the most acute toxic herbicides with the high lethality. Paraquat poisoning has become a serious public health problem in some Asian countries because of misuse or suicide^1–5^. It is estimated that there were 2,000 cases of paraquat poisoning occurred each year. Studies showed that the patient’s survival period was prolonged if fast and effective thearpy was treated. Therefore, early accurate diagnosis of paraquat poisoning is essential for the patients who intentionally concealed or the medical history was unclear^6^.

**Figure 1:**
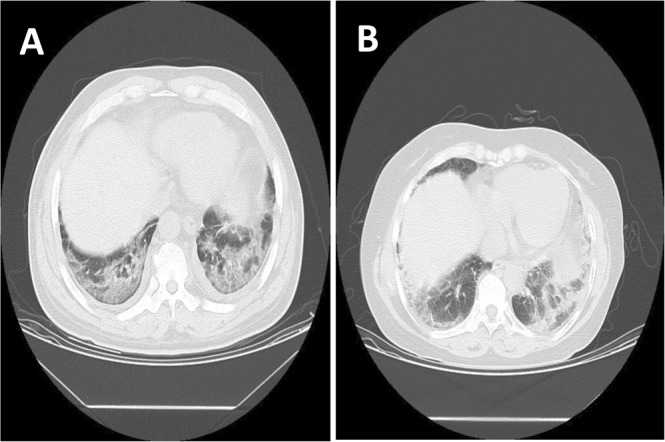
A paraquat poisoning patient (**A**) and a pneumonia patient (**B**) who were underwent CT scan.

Common clinical symptoms of paraquat poisoning include shortness of breath, chest discomfort, and occasional symptoms of cough and sputum. When the medical history was unclear or the patients intentionally concealed, clinicians often tended to look for alternative diagnoses that might explain the patient’s symptoms, of which misdiagnosed as pneumonia were most common. The CT images of pneumonia manifested as increasing internal bubble transparency, inflated bronchogram, pulmonary consolidation, local pleural thickening, pleural constriction, adjacent GGO foci, concurrent bronchial wall thickening, interlobular septal thickening, central lobe nodules and pleural effusion^7^. However, The early lung injury of paraquat poisoning was mainly an inflammatory reaction with interstitial and alveolar pulmonary edema; the CT images could also show thickening of lung texture, density of ground glass, and interstitial changes. The two have a great overlap in CT images and are difficult to be distinguished by eyes (Fig. 1).

Radiomics is an extension of computer-aided diagnosis which was presented by Lambin in 2012. It involves extracting image features and combining them with other patient data, as available, to increase the power of decision support models. Radiomics has been successfully used in the identification, staging and evaluation of lung cancer. Therefore, radiomics have the potential to identify paraquat-induced lung injury and pneumonia.

Thus, we aims to build and verify a radiomics nomogram that incorporates the radiomics features and clinical risk factors to identify paraquat poisoning lung injury and pneumonia.

## Materials and Methods

This study was approved by ethics committee of the second hospital of HeBei medical university. Due to the retrospective nature of this study, informed consent was waived.

### Patients

We collected patients with pneumonia diagnosed from November 2014 to October 2017, and acute paraquat poisoning patients who were admitted to the emergency department during the same time. Clinical information including demographic data, clinical symptoms and laboratory test results were also recorded. Data Supplement listed the inclusion and exclusion criteria and the study flow for patient enrolment^8^.

Baseline epidemiologic and clinical characteristics, including age, gender; clinical symptoms of body temperature, cough, sputum, nausea and vomiting; laboratory tests as white blood cell (WBC), creatine kinase isoenzyme (CK-MB), lactate dehydrogenase (LDH), albumin (ALB), alanine aminotransferase (ALT), aspartate transaminase (AST), Urea, creatinine (Cr), were obtained from medical records. Clinical symptoms were symptoms at the time of admission, and laboratory examinations were carried out by blood test within 3 days after admission.

Finally, 180 patients were included in the study, of which 80 were confirmed with paraquat poisoning. All 180 patients were randomly divided into two cohorts in a ratio of 7:3 using computer-generated random numbers, with 126 cases in the primary dataset and 54 cases in the validation dataset.

### CT image acquisition

Chest CT images were obtained with a GE Lightspeed/16-slice scanner. The scanning parameters were as follows: 120 kV; 100 mAs; pitch was 1.2 mm. rotation time: 0.5 s; matrix size: 512 × 512, thickness: 5.0 mm.

### Image processing

All CT images were analyzed by a free and open source ITK-SNAP software (www.itksnap.org) for semi-automatic image segmentation. Firstly, we used region growing method to sketch the whole lung as ROI, which was then manually modified by two radiologists with more than five years of experience. Data Supplement presents the ROI drawing methods and modification criteria.

### Radiomics features extraction

Analysis-Kit software (GE Healthcare, Life Science, China) was utilized to extract the radiomics features. A total of 385 radiomics features, including 42 histogram features, 154 grey level co-occurrence matrix (GLCM) features, 180 run length matrix (RLM) features and 11 grey level zone size matrix (GLZSM) features were extracted from the ROI. Details of the radiomics feature extraction methodology and the individual parameters can be found in the Supplementary Data.

### Feature extraction and radiomics signature building

In the primary cohort, we adopted the least absolute shrinkage and selection operator (LASSO) method for feature selection to filter out the effective features. Radiomics scores (Rad-scores) was calculated in each patient through a linear combination of the extracted features with their respective coefficients for prediction model.

### Validation of radiomics feature

We evaluated the ability of the radiomics feature for differentiation of pneumonia and acute paraquat lung injury in the training and validation cohort. The results were represented by the confusion matrix and the area under the curve (AUC) of the receiver operating characteristic curve (ROC).

### Development of radiomics nomogram

Multivariable backward logistic regression analysis were adopted by combination of radiomics signature and clinical candidate factors: body temperature, sputum, nausea and vomiting, AST, ALB, WBC, CK-MB and Urea using Akaike’s information criterion as the stopping rule.

A radiomics nomogram involving radiomics signature and clinical factors was built based on multivariable logistic regression analysis in the training cohort for predicting individual probability of differentiating bacterial pneumonia and acute paraquat lung injury.

### Diagnostic efficacy of the nomogram in the training cohort

The calibration curve of the nomogram mainly depends on the comparison between the actual observed forecasting ability and the standard forecasting ability. We used the AUC to evaluate the predictive power of the nomogram.

#### Independent validation

The radiomics nomogram in primary cohort was tested in the validation cohort. Each patient’s total score were calculated by the logistic regression formula. Then we performed logistic regression in the group by using the total score as a factor. Finally, calibration curve was derived based on the regression analysis.

### Clinical application

Decision curve analysis was drawn for evaluating the net benefits for clinical application of the radiomics nomogram at different probability threshold value in the validation cohort.

### Statistical analysis

Parts statistical analyses were performed by using SPSS 21.0. Chi-square test was used for the comparison of count data. Measurement data were compared by using independent samples t test if the data satisfied the normal distribution, otherwise using Mann-Whitney U test. The inter-observer correlation coefficients (ICCs) was used to assess the agreement of radiomics features by two-level radiologists. The process of using packages in R in this study were reported in the Data Supplement. *P* < 0.05 was considered statistically significant.

### Ethical approval and informed consent

The ethics committee of the second hospital of HeBei medical university approved the conducted research approved this study and the need to obtain written informed consent was waived due to its retrospective nature. The study was performed according to the principles of the declaration of Helsinki.

## Result

### Patients

**Figure 2:**
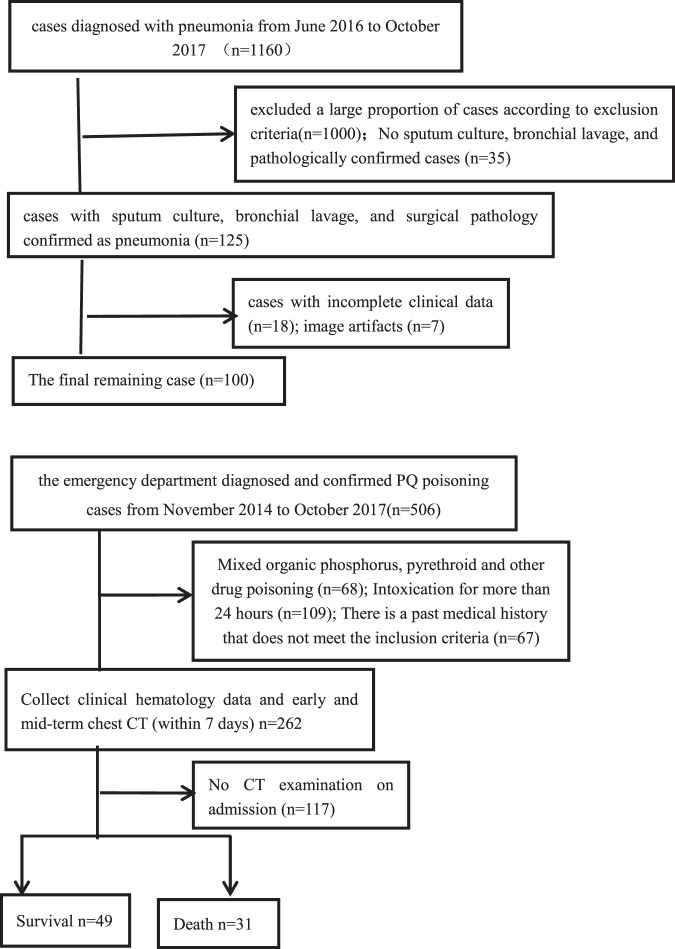
Flow chart of study enrollment of pneumonia patients and flow chart of study enrollment of paraquat poisoning patients.

Among the more than 1,000 patients with suspected pneumonia, we excluded a large proportion of cases according to exclusion criteria. Finally, 100 cases were remained in the study. Among the 506 patients of paraquat poisoning, 80 patients were included in the study according to the inclusion and exclusion criterion (Fig. 2).

Among the 180 patients in the study, we performed statistical test in demographic data, clinical features, and laboratory tests. The results are shown in Table 1. The results showed that body temperature, cough, sputum, nausea and vomiting, WBC, CK-MB, ALB, and AST were significantly different between the PQ poisoning and pneumonia.

**Table 1:** Patients with clinical data and Statistics results.

Characteristic	PQ	Pneumonia	*P* value
Number of cases	80	100	
Age	35.58 ± 12.429	39.18 ± 11.749	0.069
Sex			0.954
Female	36 (45.6)	46 (46.0)	
Male	43 (54.4)	54 (54.0)	
Symptom			
Tem (°C)	36.6 ± 0.15	38.06 ± 0.13	<0.001
Cough	1 (1.4)	75 (75.0)	<0.001
Sputum	4 (5.7)	57 (57.0)	<0.001
Sick and vomit	52 (74.3)	5 (5.0)	<0.001
Laboratory			
WBC (10^9^/L)	12.00 ± 5.80	6.90 ± 5.00	<0.001
CK-MB (U/L)	18.00 ± 10.25	14.00 ± 8.0	<0.01
LDH (U/L)	219.20 ± 55.25	225.00 ± 111.30	0.932
ALB (g/L)	17.35 ± 12.65	37.05 ± 7.83	<0.001
ALT (U/L)	22.01 ± 8.43	20.65 ± 21.55	0.984
AST (U/L)	45.54 ± 5.88	20.40 ± 13.80	<0.01
Urea (mmol/L)	4.50 ± 2.15	4.31 ± 2.31	0.641
Cr (umol/L)	70.00 ± 29.95	57.65 ± 30.38	0.056

### Feature extraction and radiomics signature building

**Figure 3:**
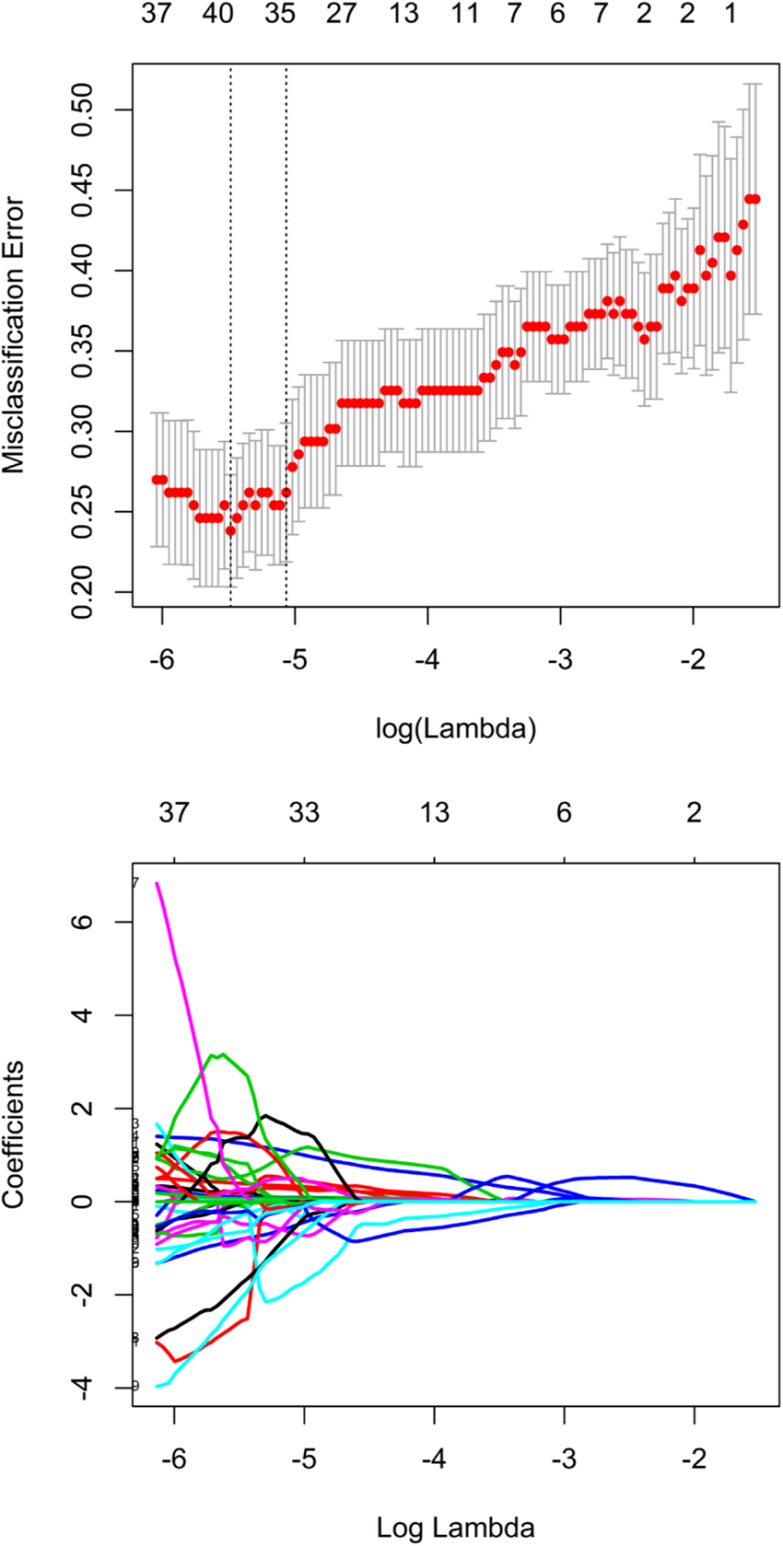
Tuning parameter λ was selected in the LASSO model using 10-fold cross-validation via minimum criteria. Misclassification error was plotted versus log(λ). The vertical line was plotted at the optimum value by using the 1 standard error and the minimum criteria.

Of the radiomics characteristics, 34 potential predictors were screened out from 385 features based on 126 patients in the training cohort, with non-zero coefficients in the LASSO logistic regression model (Fig. 3). The inter-observer correlation coefficients (ICCs) between two radiologists’ agreement is 0.823 (0.762 to 0.971, 95% CI). These features were presented in the Rad-score calculation formula. Each patient’s Rad-scores in training and validation cohorts were showed in the Supplementary Data.

### Validation of radiomics feature

**Figure 4:**
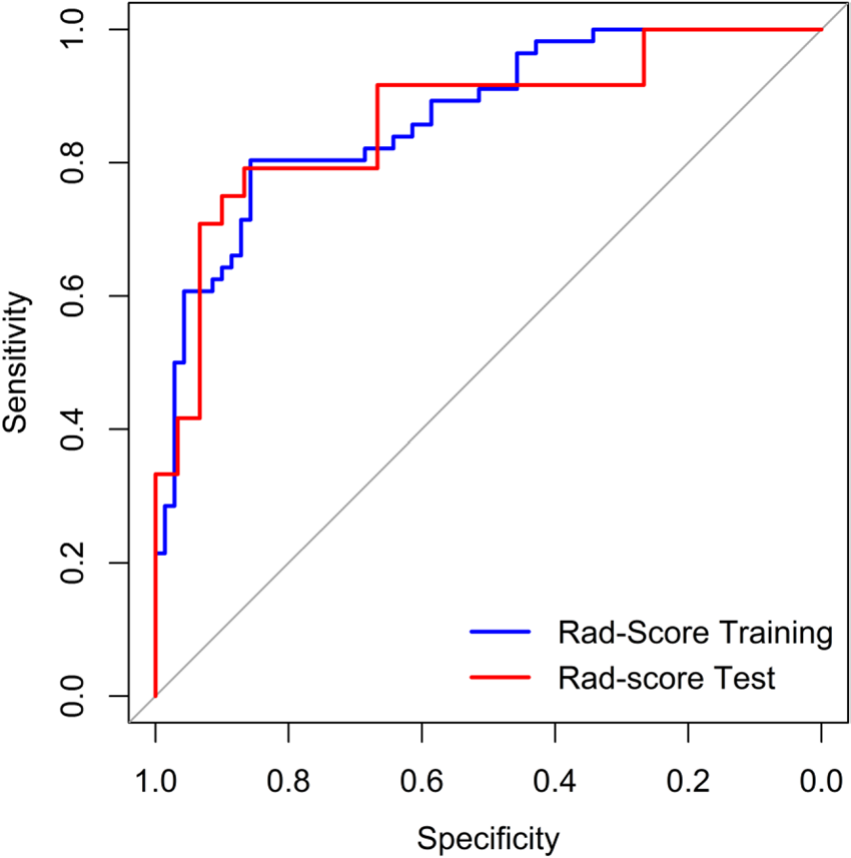
ROC curves for primary and validation cohorts.

The Rad-score between pneumonia and acute paraquat lung injury patients existed remarkable difference in the training cohort (P < 0.001), which was verified in the validation cohort (P = 0.01). The radiomics signature showed AUC 0.870 (95% CI 0.757–0.894), sensitivity 85.7%, specificity 80.4%, positive predictive value 83.3% and negative predictive value 91.4% in the primary cohort, while the validation cohort showed similar results (0.865 (95% CI 0.686–0.907), 83.3%, 79.2%, and 81.5%, and 86.7%, respectively). The ROC curves were showed in Fig. 4.

### Development of an individualized prediction model

**Figure 5:**
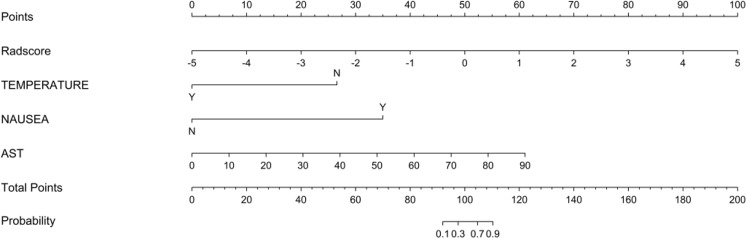
Radiomics nomograms of developed in primary cohorts. Note: The temperature was expressed in two categories, when temperature >37 was Y, and when temperature 37 was N.

We enrolled the radiomics signature, temperature, nausea and vomiting, and AST as predictors in a logistic regression analysis. The nomogram was developed by incorporating the above independent predictors (Fig. 5).

### Apparent performance of the integration between the clinical markers and radiomics in the training cohort

**Figure 6:**
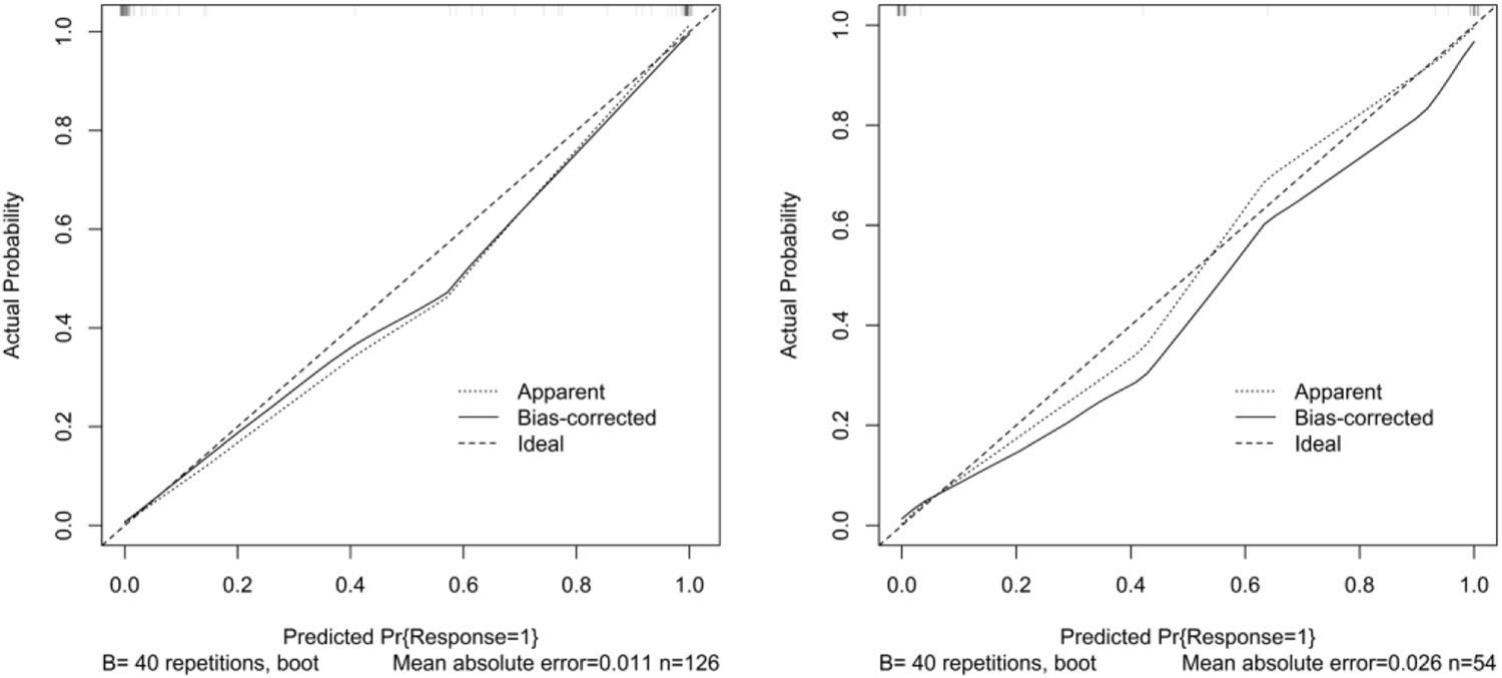
Calibration curve of radiomics nomogram. Left: calibration curve of the training cohort; Right: calibration curve of the validation cohort. The y-axis shows the actual result. The x-axis represents the predicted probability of PQ. The diagonal dotted line represents an ideal model. The solid line indicates the performance of the nomogram. If the solid line is closer to the diagonal dotted line, it means a better prediction.

**Figure 7:**
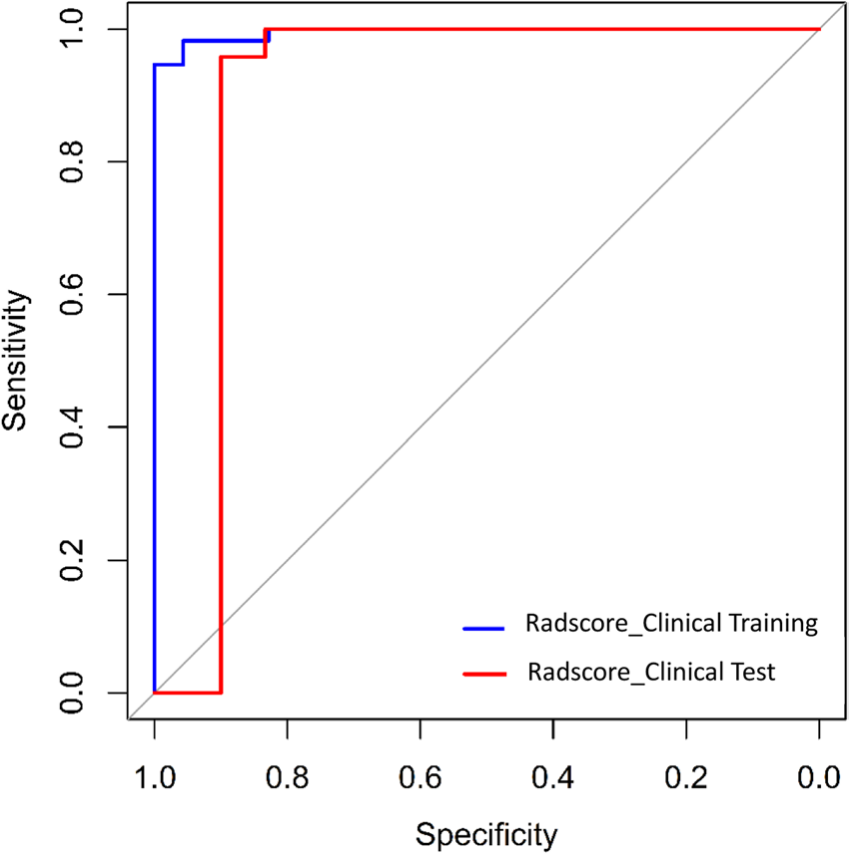
ROC curves of the integration between the clinical markers and radiomics (Radscore_Clinical) for primary and validation cohorts, each were 0.995 and 0.897.

The calibration curve of Radscore_Clinical (the integration between the clinical markers and radiomics) for the differentiation of bacterial pneumonia and acute paraquat lung injury proved good consistency between prediction and observation in the training cohort (Fig. 6). The AUC in primary cohort was 0.995, the sensitivity was 100%, and the specificity was 94.6%, with an accuracy of 97.6%. Independent verification conducted in the validation cohort also showed improved performance with above indexes being 0.897, 90.0%, 95.8%, and 92.6%, respectively (Fig. 7).

### Clinical application

**Figure 8:**
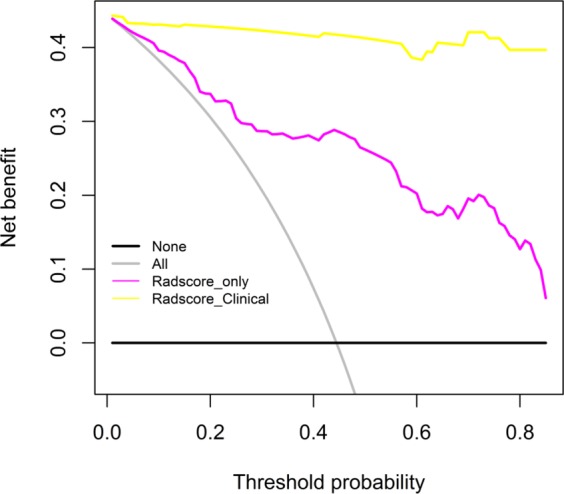
Decision curves for radiomics nomogram. The y-axis represents the net benefit (The net benefit was calculated by subtracting the proportion of all false-positive patients from the true-positive patient, and the weight is the relative hazard of abandoning treatment versus negative patients). The red dotted line indicates the radiomics nomogram. The black dotted line indicates the radiomics features. The grey solid line indicates the hypothesis that all patients were treated by one scheme (for example, assuming that all patients were with PQ poisoning). The black line represents the hypothesis that all patients were treated by another scheme (For example, assuming that all patients were with pneumonia). Obviously, the radiomics nomogram was better than Rad-score with added net benefit.

The decision curve analysis for the radiomics signature and the integrated radiomics nomogram with clinical information is showed in Fig. 8. The decision curve showed that the radiomics nomogram had significantly improved performance in the entire threshold range, compared with the radiomics signature only.

## Discussion

In the present study, we identified CT-based radiomics as a new approach for differentiating paraquat poisoning lung injury and pneumonia. To our knowledge, this was the first study of CT-based radiomics for distinguishing paraquat poisoning lung injury and pneumonia. To develop the radiomics signature, a total of 385 candidate features were reduced to a set of only 34 potential descriptors by using a LASSO logistic regression model. This method is not only superior to the method of selecting the predictor based on the univariate correlation intensity of the predictor and the outcome, but also can incorporate the panel of the selected feature into the radionomics features^9^. More importantly, we have found that the radiomics has a good discrimination performance, which demonstrated that the radiomics can be used for not only lung tumor diseases, but also for the identification of paraquat poisoning lung injury and pneumonia, providing new insights for distinguishing biological and chemical damage of lung.

Although PQ-induced irreversible pulmonary injury and pneumonia are similar in images, the mechanism of the lungs injury, the molecular and microenvironment were different. Some studies^10,11^ have shown that early pathological manifestations include pulmonary edema of varying degrees in alveolar epithelial cells and increased levels of inflammatory factors in PQ-poisoned patients. The alveolar surface was uneven and it demonstrated many cytoplasmic vesicles in type I alveolar epithelial cells earlier after intoxication. These vesicles progressively burst and released liquid content, which caused morphological changes of the alveolar cells, such as alveolar cell swelling, distortion, DNA fragmentation. Ultimately those lead to cell death. With the destruction of alveolar cells, further rupture of pulmonary capillaries leads to alveolar hemorrhage, lung infection, and pulmonary fibrosis. In the case of pneumonia, bacteria or viruses activate pulmonary epithelial cells, produce inflammatory mediators, cause damage to lung structure and epithelial cells, and lead to vacuolar degeneration of epithelial cells, mitochondrial swelling^12,13^, and subsequent intracellular vacuolization cytoplasmic distortion and cell damage^14^, further induce apoptosis of lung endothelial cells, trigger pulmonary edema and acute respiratory distress syndrome. Radiomics can extract high volumes of information from images and reflect the heterogeneity of lesions^15^. For example, GLCM features mainly reflect the internal texture of lesions. Cluster Shade feature mainly reflect the change of density inside the lesion. Therefore, different radiomics features can be extracted even for regions that are visually without lesion. This may be the root cause of distinguishing PQ poisoning lung injury and pneumonia by radiomics signature.

In this study, we collected demographic factors, clinical symptoms, and initial laboratory tests that may be related to the identification. Body temperature, nausea and vomiting, AST and radiomics signature were finally selected. On one hand, PQ patients and pneumonia patients are different in terms of temperature rise, nausea and vomiting in clinical manifestations. On the other hand, we found that some laboratory indicators are meaningful. Yang^16^ found that acute paraquat poisoning patients appeared AST elevation, the mortality rate was generally high, indicating a poor prognosis. Therefore, AST can be used as an indicator to identify PQ poisoning and pneumonia. Although PQ has direct nephrotoxicity, there is no statistical difference between urea and creatinine in this experiment. The probable reason is that the kidney has strong compensatory ability. When the glomerular filtration rate is reduced to 50% of the normal value, creatinine and urea can still be normal, and creatinine can also be affected by exogenous factors. Ralib^17^ showed that creatinine and urea were not sensitive enough in early renal injury and could not predict changes in later renal function. Although there are some differences between the ALB, Wang JL^18^ believed that the ALB in patients with PQ poisoning on the first day of hospital admission may be associated with a transient increase in blood concentration (emetic, diversion, fasting water). Therefore, the sensitivity of blood creatinine, urea nitrogen, and ALB is poor. WBC may be associated with inflammatory reactions in the lungs. The lung injury caused by PQ poisoning and pneumonia both have leukocytes and inflammatory mediators accumulated in the lungs. Therefore, WBC are elevated in both and may not be a predictor.

Image acquisition is an important part of the radiomics research. Accurate segmentation of lesions is very important for feature extraction and model construction. For different purposes, we need to segment different regions of ROIs using different segmentation methods^19^. Nowadays, doctors believed that the gold standard was the manual segmentation of lesion areas by radiologists. However, this method had the disadvantages of large individual differences, time-consuming, low efficiency, and low repetition rate. Therefore, this study chose a semi-automated method of region growing, which was a human-computer interaction segmentation method, which was simple, stable and with high efficiency. When accompanied with manual modification, it further improved the accuracy and repeatability of ROI delineation. Another, we chose the entire lung as the ROI. The ROI covered all the imaging features of PQ poisoning lung injury and pneumonia, including GGO, consolidation, thickening of Broncho vascular bundles, cystic changes, and lungs with no obvious abnormal changes. For lesion characteristics that were difficult to quantify, it was also possible to include in this study. This not only avoided the influence of subjective factors, but also can fully measure the severity and extent of lung injury.

This study has several limitations. Firstly, the pneumonia included in this study was diagnosed by sputum culture or bronchial lavage. However, we did not classify pneumonia. It may be necessary to further research the impact of different pneumonia subtypes. Secondly, the data for this study originated from the same institution and required more research institutions to perform validation and follow-up studies. Thirdly, we did not explain the biological interpretation of radiomics features in this study completely^20^, so we are fully aware of the need for further exploration in subsequent studies.

In conclusion, a radiomics nomogram was built that integrates both the radiomics signature and clinical risk factors, and can be conveniently used to differentiate pneumonia and acute paraquat lung injury.

## Supplementary information

Supplementary data

## Data Availability

Inclusion and exclusion criteria and radiomics feature extraction methodology are available in Supplementary Data.
