# Supplementary material for: Radiomics Nomogram Analyses for Differentiating Pneumonia and Acute Paraquat Lung Injury
Source: Sci Rep. 2019 Oct 21;9:15029. doi: 10.1038/s41598-019-50886-7 (PMC6803642; doi:10.1038/s41598-019-50886-7)
Supplement: Supplementary file 1 — Supplementary data [file 41598_2019_50886_MOESM1_ESM.docx]

**Radiomics Nomogram Analyses for Differentiating Pneumonia and Acute** **Paraquat Lung Injury**

**Wang Yanling^#1^, Gao Duo^#1^, Geng Zuojun^*1^, Shi Zhongqiang^2^,** **Wu Yankai^1^, Lu Shan^1^, Cui Hongying^1^**

^1^Department of Medical Imaging, Second Hospital of Hebei Medical University, Shijiazhuang 050000, China

^2^ GE Healthcare, Shanghai, 210000, China

**# These authors contributed equally to this work.**

Wang Yanling E-mail: 13930113753@163.com

Gao Duo E-mail: gaoduo@bjmu.edu.cn

Shi Zhongqiang E-mail: llfszq_tg@163.com

Wu Yankai E-mail: 1061382709@qq.com

Lu Shan E-mail: lushandf@qq.com

Cui Hongying E-mail: 710431964@qq.com

***Corresponding Author:**

Prof. Geng Zuojun, Department of Medical Imaging, Second Hospital of Hebei Medical University, Shijiazhuang, China

Postal address: 050000

E-mail address: [1980756261@qq.com](javascript:;)

Work telephone number: 0311-66003789

Work fax number: 0311-66003789

**Appendix A1:** **Inclusion and exclusion criteria**

Inclusion criteria for patients with paraquat poisoning:

1. Paraquat poisoning diagnosis was clear which conformed the Chinese Medical Association emergency physicians branch "acute paraquat poisoning diagnosis and treatment expert consensus (2013)" paraquat poisoning diagnostic criteria;
2. came to the emergency department within 24 hours;
3. Completed poison inspections, initial blood biochemical tests, and completed images and clinical data;
4. no significant other non-toxicity change in lung images.

Exclusion criteria:

1. other drugs or pesticide poisoning;
2. a history of severe lung disease such as persistent lung infection or recent infectious disease；
3. having past pulmonary tuberculosis, idiopathic pulmonary fibrosis or severe liver, kidney, blood system and other serious diseases;
4. having been treated in our hospital for more than 24 h;

Inclusion criteria for patients with pneumonia:

1. patient who meets the diagnostic criteria established by the 2007 ATS and the American Society of Infectious Diseases (IDSA), and be confirmed by blood culture, sputum culture or bronchial lavage；
2. complete image and clinical data
3. performed CT examination in our hospital within 3 days of onset.

Exclusion criteria:

1. a history of severe lung disease such as persistent lung infection or recent infectious disease；
2. typical pneumonia such as lobar pneumonia which have significant different image appearance with paraquat poisoning；
3. pneumonia represented mainly as consolidation or pleural effusion；
4. patient without chest CT examination within 3 days of onset.

**Appendix A2: ROI drawing methods and modification criteria.**

The region growing method is mainly divided into three steps :1 The seed points were selected from the seed area that can represent the extraction area, and the seed was a small area including a couple of pixels; 2 Determine the criteria for region growing, and measure whether the pixels adjacent to the seed point meet the criteria. The standards outlined in this study were: Lower threshold was -1200Hu, Upper threshold was -100Hu; 3 Stop growing . After region growing, the boundary between the apex of the lung and the edge of the lung needed manual modification.

The criteria of manual modification were as follows：1) The boundary between the chest wall and the lung: the lesion-free areas were automatically outlined without modification; those areas with lesion but the lesion was not totally included in the ROI were manually modified. 2) If the demarcation of lung atelectasis caused by pleural effusion was unclear, automatic delineation results was used without manual modification. 3) For the higher density of lung lesions, such as cords and nodules which were not covered in the ROI, manual delineation was applied. 4) For lung lesions which were not included in the ROI automatically, manual delineation was applied. 5) Vascular and bronchi: The ROI contained no main and leaf bronchi; If the segmental and inferior bronchi were connected to pixels that were distinguishable by the naked eye, we didn’t not sketch them into the ROI. Otherwise, we sketched them into the ROI; The small scattered bronchus of lungs was contained in the ROI. 6) For those lesions with poorly borderline in hilum, the principle was not missing lesions as far as possible. 7) For apex and bottom of lung: Slices without lung tissue was removed manually; Slices with lung tissue but only scattered pixels in the border were included, we modified the ROI to the edge of the lung tissue manually.

**Appendix A3: The packages of R software used for statistical analysis**

Feature selection and model building were conducted with R software (version 3.3.2; http://www.Rproject.org). The packages in R that were used in this study were as follows: Lasso binary logistic regression was done using the “glmnet” package. Multivariate binary logistic regression, nomograms and calibration plots were done with the “rms” package. PCA was done using the "stats" package. ROC (Receiver operating characteristics) curve was plotted using the “pROC” package. Nomograms and calibration plots were done with the “rms” package. Decision curve analysis was performed with the function of “dca.R”.

**Appendix A4: Radiomics feature extraction methodology**

In our study, a total of 385 imaging features for each patient were extracted. All radiomics features were calculated automatically with the noncommercial A.K. (Analysis Kit) software (GE Healthcare, China). The 385 features were divided into three types: first-order: histogram parameters; second order texture features: gray-level co-occurrence matrix (GLCM), gray-level run length matrix (GLRL) features and gray-level size zone matrix (GLSZM) features and form factor features

**Group1: First-Order:** **histogram parameters**

**1. Minimum Intensity:** The value of the voxel(s) in the image ROI with the least value. **2.** **Maximum Intensity:** The value of the voxel(s) in the image ROI with the greatest value.

**3. Median Intensity:** The median of the intensity or parameter values within the image ROI.

**4. Mean Intensity:** The mean of the intensity or parameter values within the image ROI.

$mean=\frac{1}{N}\sum_{i}^{N} X(i)$

**5. Standard Deviation:** Measures the amount of variation or dispersion from the mean of the values in the image ROI.

$s\tan dard deviation=\left(\frac{1}{N-1}\sum_{i=1}^{N} (X(i)-\bar{X})^{2}\right)^{1/2}$

**6.** **Variance:** The mean of the squared distances of each value in the image ROI from the mean of the values. This is a measure of the spread of the distribution about the mean.

$\mathrm{var}iance=\frac{1}{N-1}\sum_{i=1}^{N} (X(i)-\bar{X})^{2}$

**7. Voxel Count:** The total number of voxels within the ROI of the grayscale image or parameter map. Describe the size of the ROI.

**8.** **Voxel Value Sum**: The sum of voxels within the ROI of the grayscale image or parameter map.

**9.** **Range:** The difference between the highest and lowest voxel values within the image ROI.

**10.** **Root Mean Square:** The square-root of the mean of the squares of the values in the image ROI. It is another measure of the magnitude of the image values.

$RMS=\sqrt{\frac{\sum_{i}^{N}X(i)^{2}}{N}}$

**11.** **Mean Deviation:** The mean of the distances of each image value from the mean of all the values in the image ROI.

**12. Relative Deviation:** Let $\bar{X}$denote the mean of a set of quantities$X_{i}$, then the relative deviation is defined by:

$\frac{\Delta X_{i}}{\bar{X}}=\frac{|X_{i}-\bar{X}|}{\bar{X}}$

**13.** **Skewness:** Measures the asymmetry of the distribution of values in the image ROI about the mean of the values. Depending on where the tail is elongated and the mass of the distribution is concentrated, this value can be positive or negative.

$skewness=\frac{\frac{1}{N}\sum_{i=1}^{N}(X(i)-\bar{X})^{2}}{\left(\sqrt{\frac{1}{N}\sum_{i=1}^{N}(X(i)-\bar{X})^{2}}\right)^{2}}$

**14. Kurtosis:** A measure of the 'peakedness' of the distribution of values in the image ROI. A higher kurtosis implies that the mass of the distribution is concentrated towards the tail(s) rather than towards the mean. A lower kurtosis implies the reverse, that the mass of the distribution is concentrated towards a spike the mean.

$kurtosis=\frac{\frac{1}{N}\sum_{i=1}^{N}(X(i)-\bar{X})^{4}}{\left(\sqrt{\frac{1}{N}\sum_{i=1}^{N}(X(i)-\bar{X})^{2}}\right)^{2}}$

**15. Uniformity:** A measure of the sum of the squares of each discrete value in the image ROI. This is a measure of the heterogeneity of an image, where a greater uniformity implies a greater heterogeneity or a greater range of discrete image values.

$uniformity=\sum_{i=1}^{N_{l}} P(i)^{2}$

**16. Energy:** A measure of the magnitude of values in an image. A greater amount larger values implies a greater sum of the squares of these values.

$energy=\sum_{i}^{N} X(i)^{2}$

**17. Entropy:** Specifies the uncertainty in the image values. It measures the average amount of information required to encode the image values.

$entropy=-\sum_{i=1}^{N_{l}} P(i)\log_{2}P(i)$

**18. Frequency Size**

**19-37. Percentiles：**A percentile is a measure used in statistics indicating the value below which a given percentage of observations in a group of observations fall. There was 19 percentiles from Percentile5 to Percentile95 with the interval of 5.

**38-42. Quantiles：** For a finite population of *N* equally probable values indexed 1, …, $N$ from lowest to highest, the $k$-th $q$-quantile of this population can equivalently be computed via the value of:$\mathbf{Ip}=\text{ }\mathbf{N}\text{ }\mathbf{k}/\mathbf{q}$**.** Here, we have 5 quantiles, including Quantile0.025, Quantile0.25, Quantile0.5, Quantile0.75, Quantile0.975.

**Group2 Second Order Texture Features**

While first order statistics describe the distribution of the gray values within an image, they do not contain information about the texture of a given region. This can be done by using gray-level co-occurrence matrix (GLCM), ray-level run length matrix (GLRLM) features, and gray-level size zone matrix (GLSZM) features.

**2.1 Gray-Level Co-occurrence Matrix (GLCM)**

The Grey level co-occurrence matrix (GLCM) $\Pr(i,j|\theta,d)$represents the joint probability of certain sets of pixels having certain grey-level values. It calculates how many times a pixel with grey-level **i** occurs jointly with another pixel having a grey value **j**. By varying the displacement vector **d** between each pair of pixels. Here, we have 18 parameters related to each of the following 8 GLCM features.

**43-60**. **Energy of GLCM**

Also known as the Angular Second Moment and is a measure of the homogeneity of an image. A homogeneous image will contain less discrete gray levels, producing a GLCM with fewer but relatively greater values of P(i,j), and a greater sum of the squares.

$\sum_{i,j}^{(Formula)} g(i,j)^{2}$

$^{*}g$is a GLCM

Where $i,j$ are the spatial coordinates of$(i,j)$

The 18 parameters related to the GLCM Energy: GLCMEnergy_AllDirection_offset1, GLCMEnergy_AllDirection_offset1_SD, GLCGLCMEnergy_angle45_offset1, GLCMEnergy_angle90_offset1, GLCMEnergy_angle135_offset1, GLCMEnergy_AllDirection_offset4, GLCMEnergy_angle0_offset4, GLCMEnergy_angle45_offset4, GLCMEnergy_angle90_offset4, GLCMEnergy_angle135_offset4, GLCMEnergy_AllDirection_offset4_SD, GLCMEnergy_AllDirection_offset7, GLCMEnergy_angle0_offset7, GLCMEnergy_angle45_offset7, GLCMEnergy_angle90_offset7, GLCMEnergy_angle135_offset7, GLCMEnergy_AllDirection_offset7_SDMEnergy_angle0_offset1,

**61-78. Entropy of GLCM:** Indicates the uncertainty of the GLCM. It measures the average amount of information required to encode the image values.

$-\sum_{i,j}^{Formula} g(i,j)\log_{2}(i,j)$

**79-96. Inertia of GLCM:** It reflects the clarity of the image and texture groove depth. The contrast is proportional to the texture groove, high values of the groove produces more clarity, in contrast small values of the groove will result in small contrast and fuzzy image.

$\sum_{i,j}^{Formula} ((i,j)^{2}\mathrm{g}(i,j))$

**97-114. Correlation:** Image-based Correlation measures the similarity of the grey levels in neighboring pixels, tells how correlated a pixel is to its neighbor over the whole image. Range = [-1 1]. Correlation is 1 or -1 for a perfectly positively or negatively correlated image.

$-\sum_{i,j}^{Formula} \frac{(i-\mu)(j-\mu)g(i,j)}{\sigma^{2}}$

**115-132. Inverse Difference Moment (IDM):** IDM is the local homogeneity. It is high when local gray level is uniform and inverse GLCM is high. IDM weight value is the inverse of the Contrast weight.

$\mathop{\sum\sum}\limits^{Formula} \frac{1}{1+(i-j)^{2}}g(i,j)$

**133-150. Cluster Shade:** A measure of the skewness and uniformity of the GLCM. A higher cluster shade implies greater asymmetry.

**151-168.** **Cluster Prominence:** A measure of the skewness and asymmetry of the GLCM. A higher value implies more asymmetry about the mean value while a lower value indicates a peak around the mean value and less variation about the mean.

$\sum_{i,j}^{Formula} ((i-\mu)+(j-\mu))^{4}g(i,j)$

**Haralick features**

**169-186.** **Haralick Correlation:** Measures the degree of similarity of the gray level of the image in the row or column direction. Represents the local grey level correlation, the greater its value, the greater the correlation;

$-\sum_{i,j}^{Formula} \frac{(i,j)g(i,j)-\mu_{t}^{2}}{\sigma_{t}^{2}}$

* where μ*_t_* and σ*_t_* are the mean and standard deviation of the row (or column, due to symmetry) sums.

**187. Angular Second Moment(ASM)**

$f_{1}=\sum_{i=1}^{N_{g}} \sum_{j=1}^{N_{g}} \left(\frac{P(i,j)}{R}\right)^{2}=\sum_{i} \sum_{j} p(i,j)^{2}$

**188. Contrast:** The contrast feature, is a difference moment of the P matrix and is a measure of the contrast or the amount of local variations present in the image.

$f_{2}=\sum_{k=0}^{N_{g}-1} k^{2}\left\{\sum_{i=1}^{N_{g}} \sum_{j=1}^{N_{g}} \delta_{\left|i-j\right|,k}p(i,j)\right\}=\sum_{k=0}^{N_{g}-1} k^{2}p_{x-y}(k)$

**189. HaraVariance**

$f_{4}=\sum_{i=1}^{N_{g}} \sum_{j=1}^{N_{g}} (i-\mu)^{2}p(i,j)$

**190. inverse Difference Moment**

$f_{5}=\sum_{i=1}^{N_{g}} \sum_{j=1}^{N_{g}} \frac{1}{1+(i-j)^{2}}p(i,j)$

**191. Sum Average**

$f_{6}=\sum_{i=2}^{2N_{g}} ip_{x+y}(i)$

**192. Sum Variance**

$f_{7}=\sum_{i=2}^{2N_{g}} (i-f_{8})^{2}p_{x+y}(i)$

**193. Sum Entropy**

$f_{8}=-\sum_{i=2}^{2N_{g}} p_{x+y}(i)\log(p_{x+y}(i))$

**194. Haralick Entropy**

$f_{9}=-\sum_{i=1}^{N_{g}} \sum_{j=1}^{N_{g}} p(i,j)\log(p(i,j))$

**195.Difference Variance**

$f_{10}=variance of p_{x-y}$

**196.Difference Entropy**

$f_{11}=-\sum_{i=0}^{N_{g}-1} p_{x-y}(i)\log(p_{x-y}(i))$

**Gray-Level Run Length Matrix (GLRLM)**

The grey level run-length matrix (RLM) **𝐏𝐫(i, j** **|θ)** is defined as the numbers of runs with pixels of gray level *i* and run length *j* for a given direction θ. RLMs is generated for each sample image segment having directions (0°,45°,90° &135°), then ten statistical features were derived, with 18 parameters related to each of these GLRLM features, similar to GLCM.

**197-214. Short Run Emphasis (SRE):** A measure of the distribution of short run lengths, with a greater value indicative of shorter run lengths and more fine textural textures.

$SRE(\theta)=\frac{1}{n_{r}}\sum_{i=1}^{M} \sum_{j=1}^{N} \frac{p(i,j,\theta)}{j^{2}}$

**215-232. Long Run Emphasis (LRE):** A measure of the distribution of long run lengths, with a greater value indicative of longer run lengths and more coarse structural textures.

$LRE(\theta)=\frac{1}{n_{r}}\sum_{i=1}^{M} \sum_{j=1}^{N} p(i,j,\theta)j^{2}$

**233-250. Gray Level Non-uniformity (GLN):** Measures the similarity of gray-level intensity values in the image, where a lower GLN value correlates with a greater similarity in intensity values.

$$GLN(\theta)=\frac{1}{n_{r}}\sum_{i=1}^{M} (\sum_{j=1}^{N} p(i,j,\theta))^{2}$$

**251-268.Run Length Non-uniformity (RLN):** Measures the similarity of run lengthsthroughout the image, with a lower value indicating more homogeneity among run lengths in the image.

$RLN(\theta)=\frac{1}{n_{r}}\sum_{j=i}^{N} (\sum_{i=1}^{M} p(i,j,\theta))^{2}$

**269-286. Low Gray Level Run Emphasis (LGRE):** Measures the distribution of low gray-level values, with a higher value indicating a greater concentration of low gray level values in the image.

$LGRE(\theta)=\frac{1}{n}\sum_{j=i}^{N} \sum_{i=1}^{M} \frac{p(i,j,\theta)}{i^{2}}$

**287-304.High Gray Level Run Emphasis (HGRE):** Measures the distribution of the higher gray-level values, with a higher value indicating a greater concentration of high gray-level values in the image.

$HGRE(\theta)=\frac{1}{n_{r}}\sum_{j=i}^{N} \sum_{i=1}^{M} p(i,j,\theta)i^{2}$

**305-322. Short Run Low Gray Level Emphasis (SRLGE):** Measures the joint distribution of shorter run lengths with lower gray-level values.

$SRLGE(\theta)=\frac{1}{n_{r}}\sum_{j=i}^{N} \sum_{i=1}^{M} \frac{p(i,j,\theta)}{i^{2}j^{2}}$

**323-340. Short Run High Gray Level Emphasis (SRHGE):** Measures the joint distribution of shorter run lengths with higher gray-level values.

$SRHGE(\theta)=\frac{1}{n_{r}}\sum_{j=i}^{N} \sum_{i=1}^{M} \frac{p(i,j,\theta)t^{2}}{j^{2}}$

**341-358.Long Run Low Gray Level Emphasis (LRLGE):** Measures the joint distribution of long run lengths with lower gray-level values.

$LRLGE(\theta)=\frac{1}{n_{r}}\sum_{j=i}^{N} \sum_{i=1}^{M} \frac{p(i,j,\theta)j^{2}}{t^{2}}$

**359-376. Long Run High Gray Level Emphasis (LRHGE):** Measures the joint distribution of long run lengths with higher gray-level values.

$LRHGE(\theta)=\frac{1}{n_{r}}\sum_{j=i}^{N} \sum_{i=1}^{M} p(i,j,\theta)i^{2}j^{2}$

**Group3: Form factor parameters**These group of features includes descriptors of the three-dimensional size and shape of the tumor region. Let in the following definitions ***V*** denote the volume and ***A*** the surface area of the volume of interest. We determined the following shape and size based features:

**377. Sphericity**

$sphericity=\frac{\pi^{\frac{1}{3}}(6V)^{\frac{2}{3}}}{A}$

**378.Surface area**

$A=\sum_{i=1}^{N} \frac{1}{2}|a_{i}b_{i}\times a_{i}c_{i}|$

**379.Compactness 1**

$compactness 1=\frac{V}{\sqrt{\pi}A^{\frac{2}{3}}}$

**380.Compactness 2**

$compactness 2=36\pi\frac{V^{2}}{A^{3}}$

**381.Maximum 3D diameter**

**382.Spherical disproportion**

$spherical disproportion=\frac{A}{4\pi R^{2}}$

**383. Surface to volume ratio**

$surface to volume ratio =\frac{A}{V}$

**384-385. VolumeCC and VolumeMM**

**Appendix Figure A1.** Patient enrollment flow chart

cases diagnosed with pneumonia from June 2016 to October 2017 （n=1160）

excluded a large proportion of cases according to exclusion criteria(n=1000)**；**No sputum culture, bronchial lavage, and pathologically confirmed cases (n=35)

cases with sputum culture, bronchial lavage, and surgical pathology confirmed as pneumonia (n=125)

cases with incomplete clinical data (n=18); image artifacts (n=7)

The final remaining case (n=100)

the emergency department diagnosed and confirmed PQ poisoning cases from November 2014 to October 2017(n=506)

Mixed organic phosphorus, pyrethroid and other drug poisoning (n=68); Intoxication for more than 24 hours (n=74);There is a past medical history that does not meet the inclusion criteria (n=67)；bloodsystem and other serious diseased(35)

Collect clinical hematology data and early and mid-term chest CT (within 7 days) n=262

No CT examination on admission (n=117)

**30天内失去随访病例（n=65）**

Survival n=49

Death n=31

**Appendix Figure A1.** Patient enrollment flow chart

**
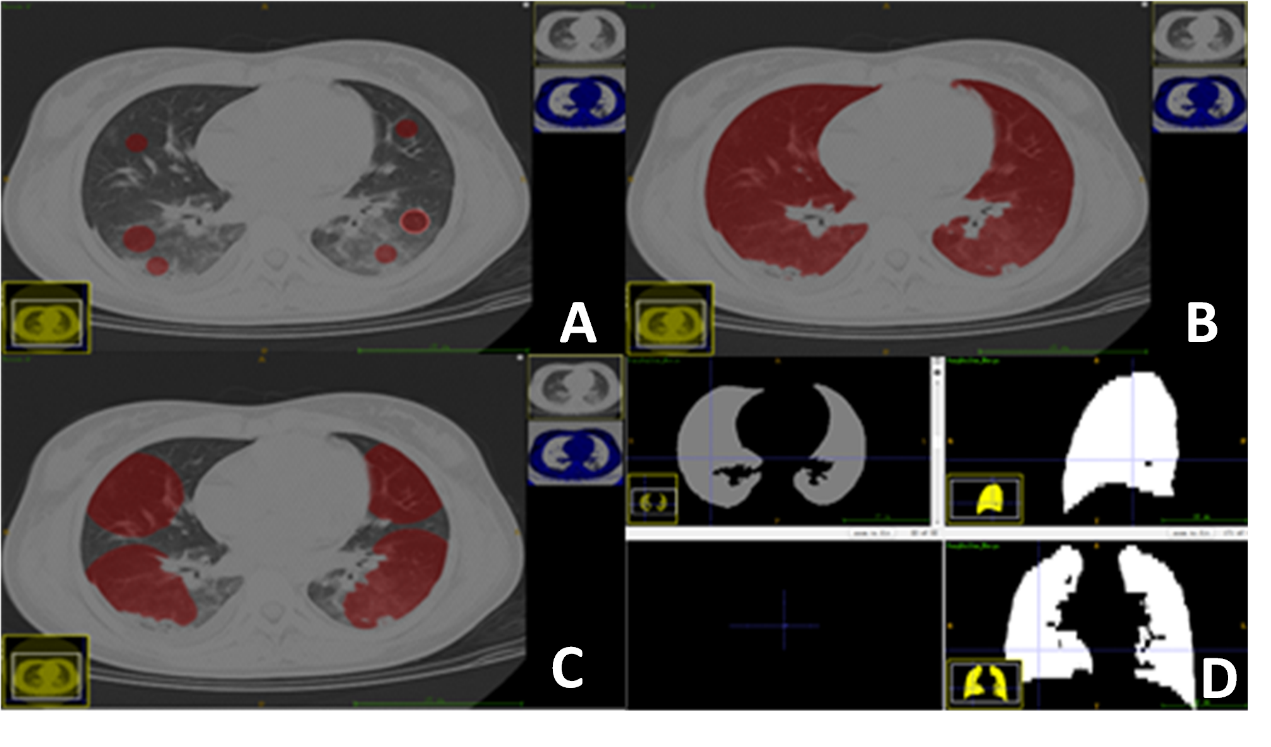
Appendix Figure A2.** Image segmentation diagram. A：Seed points were selected in three higher density lesions, lower density lesions and normal lung tissue in both lungs; B：The seed points began to grow; C：Growth of seed points completed basically; D：ROI obtained after manual modification.


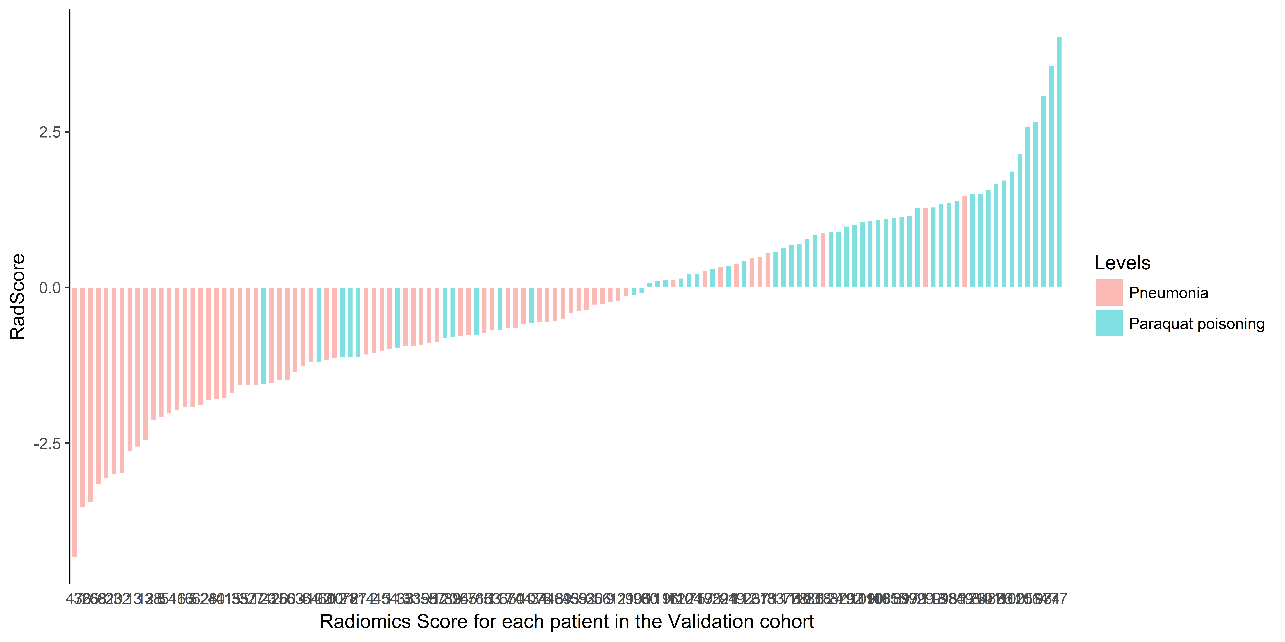
**
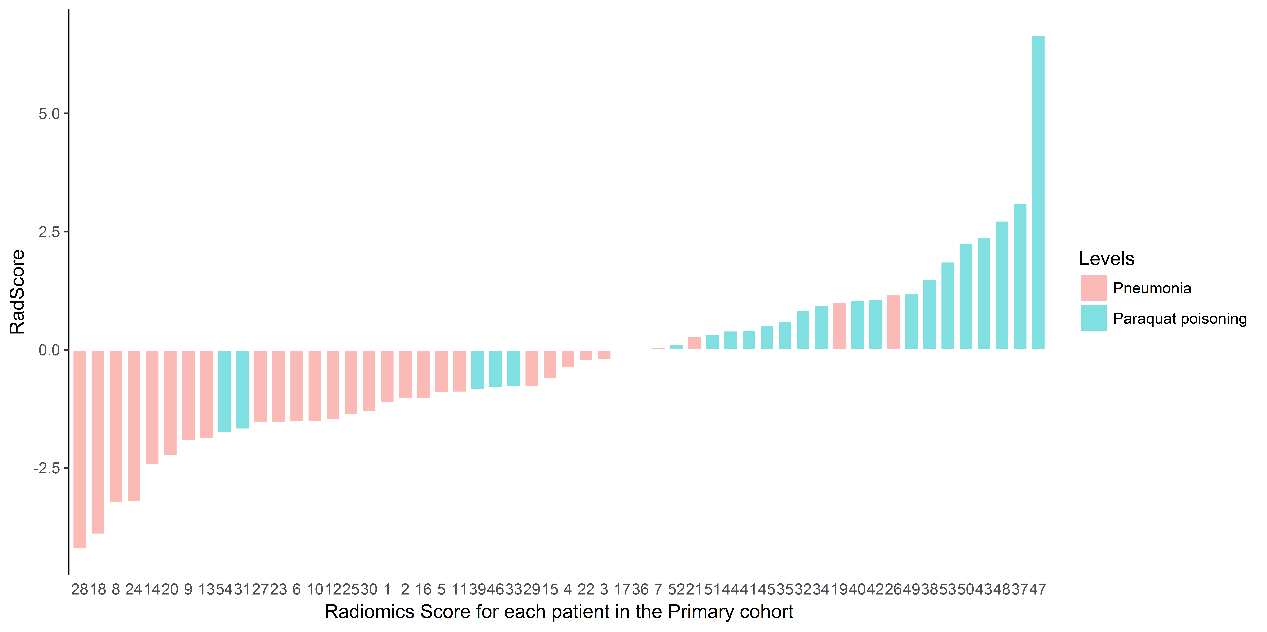
Appendix Figure A3.** Rad-score for every patient in each cohort. (A). Rad-score for every patient in the primary cohort; (B). Rad-score for every patient in the validation cohort. The status of prognosis was marked with different colors.
